# Preferred Place of Death in Adult Cancer Patients: A Systematic Review and Meta-Analysis

**DOI:** 10.3389/fpsyg.2021.704590

**Published:** 2021-08-27

**Authors:** Armin Fereidouni, Maryam Rassouli, Mahmood Salesi, Hadis Ashrafizadeh, Amir Vahedian-Azimi, Salman Barasteh

**Affiliations:** ^1^Medicine, Quran and Hadith Research Center, Marine Medicine Research Center, Baqiyatallah University of Medical Sciences, Tehran, Iran; ^2^Cancer Research Center, Shahid Beheshti University of Medical Sciences, Tehran, Iran; ^3^Chemical Injuries Research Center, Systems Biology and Poisonings Institute, Baqiyatallah University of Medical Sciences, Tehran, Iran; ^4^Student Research Committee, Nursing and Midwifery School, Ahvaz Jundishapur University of Medical Sciences, Ahvaz, Iran; ^5^Trauma Research Center, Nursing Faculty, Baqiyatallah University of Medical Sciences, Tehran, Iran; ^6^Health Management Research Center, Nursing Faculty, Baqiyatallah University of Medical Sciences, Tehran, Iran

**Keywords:** palliative care, end of life, cancer, hospice, preferred place of death, terminally ill, hospital, supportive care

## Abstract

**Background:** Identifying the preferred place of death is a key indicator of the quality of death in cancer patients and one of the most important issues for health service policymakers. This study was done to determine the preferred place of death and the factors affecting it for adult patients with cancer.

**Methods:** In this systematic review and meta-analysis study four online databases (PubMed, Scopus, web of science, ProQuest) were searched by relevant keywords. Quality assessment of papers was conducted using Newcastle-Ottawa (NOS) criterion. Odds ratios, relative risks, and 95% confidence intervals were determined for each of the factors extracted from the investigations.

**Results:** A total of 14,920 participants of 27 studies were included into the meta-analysis. Based on the results, 55% of cancer patients with a confidence interval [95% CI (41–49)] preferred home, 17% of patients with a confidence interval [95% CI (−12%) 23)] preferred hospital and 10% of patients with confidence interval [95% CI (13–18)] preferred hospices as their favored place to die. Effective factors were also reported in the form of demographic characteristics, disease-related factors and psychosocial factors.

**Conclusions:** This study showed that more than half of cancer patients chose home as their preferred place of death. Therefore, guided policies need to ensure that the death of the patients in the preferred place should be considered with priority.

**Systematic Review Registration:**https://www.crd.york.ac.uk/prospero/display_record.php?ID=CRD42020218680, identifier: CRD42020218680.

## Introduction

Cancer is considered the second leading cause of death worldwide. In 2018, about 9.6 million people of the world have died by cancer, which was almost one in six worldwide deaths (WHO, 2021). Of these, approximately 1.6 million patients did not die in their preferred place (Kern et al., 2020). Patients with advanced cancer show that despite the fact that the majority of cancer patients prefer to die in the preferred place (Neergaard et al., 2011; Hyun et al., 2013; Gomes et al., 2015; Vidal et al., 2020), a small number of these patients die in the preferred place (Chen et al., 2014; Howell et al., 2017).

The place of death has passed through three evolutionary periods in societies. In the first period, people often died at home due to poor access to health resources. Secondly, deaths in hospitals have increased due to advances in the health system, but in the last decade, the emphasis has changed to the quality of end-of-life care, and by developing home care, it has been emphasized to people's preferences to die at home. During different periods, social norms play a role in determining the place of death (Gu et al., 2007).

The place of death is very important in the allocation of medical resources and has recently received a lot of attention from palliative care specialists (Hyun et al., 2013; Cabañero-Martínez et al., 2019). In recent decades, this place has been a hospital for the majority of cancer patients (Alonso-Babarro et al., 2011; Gomes et al., 2015). In fact, the actual place of death is 10 to 35% of patients at home and 50 to 60% occurs in the hospital (Bell et al., 2010).

The preferred place of death (PPOD) means the desire of people to die in one place or death of people in a favorite place (Yamagishi et al., 2012). Over the last few decades, the ability to select and control the PPOD has increasingly been considered as a key indicator for increasing the quality of death and one of the criteria of a good death (Ali et al., 2019); Therefore, health care policymakers attribute great importance to determining PPOD. Focusing on PPOD increases the quality of end-of-life care, respect for patients' preferences, as well as proper distribution of health and medical resources to successfully implement palliative care (Gu et al., 2015; Ali et al., 2019). In a systematic review, Bell et al. studied the correlation between the preferred and actual place of death (Bell et al., 2010). Gomes et al. (2013) In a systematic review of meta-analysis, reveal that not only the majority of cancer patients but also other patients with life-threatening issues choose the home as their preferred place of death. The use of home-based end-of-life care has very beneficial effects on the physical, psychological, social, and economic dimensions of patients. Also it reduces the costs of the health system and hospital complications, shortens the length of hospitalization, and prevents re-hospitalization of patients. Also, this method of care can facilitate the continuity of care after discharge and patients benefit from the facilities of different centers (Kerr et al., 2014; Lustbader et al., 2017; Heydari, 2018). However, the home is not always the best place to die because access to home-based palliative care and care services varies from country to country (Chen et al., 2014). Due to the different PPOD, the present study was conducted to determine the PPOD and the factors affecting it in adult patients with cancer.

## Methods

### Study Design

This systematic Review study was performed based on the guidelines of Preferred Reporting Items for Systematic Reviews and Meta-Analyzes (PRISMA) (Liberati et al., 2009). Also was approved by the ethics committee of Baqiyatallah University of Medical Sciences (Ethics code: IR.BMSU.REC.1399.425). The study protocol is registered in PROSPERO with the code CRD42020218680 (the access link:https://www.crd.york.ac.uk/prospero/display_record.php?ID=CRD42020218680).

### Type of Study and Participants

Observational studies including cross-sectional, case-control, cohort that explicitly state the PPOD of cancer patients or provide data to calculate this index have been selected. Review articles, case reports, case series, and clinical trials were not included. The abstracts of studies published at conferences, case studies, reviews, qualitative studies, gray studies, and letter to the editor due to lack of use of primary data (PPOD) separately were excluded. Also to accurately and without bias determine the patient's preferences studies focusing on pediatric cancer patients (individuals under 18 years of age), presenting secondary and tertiary by nurses, physicians or family caregivers, and inability to differentiate reported outcomes for cancer patients were excluded. Primary studies were performed on cancer patients of any race, ethnicity, and one of the two sex groups of men or women or both of them, were entered into the study.

### Sampling Method and Sample Size

Sampling methods in studies were randomly systematic review (probable) (simple random sampling, systematic random sampling, stratified random sampling, cluster random sampling) or primary studies using non-random (non-probable) sampling methods (quota sampling), convenience sampling, purposive sampling, self-selection sampling, and snowball sampling) or public call announcements or a combination of them were entered into the study.

### Selection Criteria

Original articles published in English without time-limitation were identified according to search criteria's. Duplicate sources were removed using EndNote X8 software. In the screening stage, the titles and abstracts of the articles were reviewed. Selected studies were divided into three categories: related, unrelated, and unreliable. Articles reported by both unrelated researchers were excluded from the study. Then in the selection stage, the full text of the articles independently were investigated by two researchers (A.F. and S.B.). All disagreements at any stage were resolved by discussion and agreement between the two researchers. In case of disagreement between the researchers, a third person was used as a judge and the result was reported as a statistical Kappa coefficient after general agreement. Data extraction and quality assessment studies were performed by two researchers (A.F and S.B.).

### Search Strategy

The studies were searched in four databases: PubMed/Medline, Scopus, web of science, and ProQuest on October 22, 2020, without any time-limitation. The keywords for this systematic study were a combination of Mesh Term and Free Text words (Table 1). In the case of encountering a study in accordance with the objectives of the study, in the case of lack of access to the full text of articles, unpublished data or the existence of erroneous and ambiguous data, an email was sent to the corresponding author of the article and three more emails were sent at intervals of 1–10 days. If no message was received from the author of the article after 3 emails, the article was deleted. Any disagreement was resolved by agreement of the two researchers (S.B, A.F) and in case of disagreement, the opinion of the third informed person was the criterion for decision making.

**Table 1:** Search strategy.

Search engines and databases: PubMed, Scopus, web of science, ProQuest
Limits: Language (Only resource with at least an abstract English)
Date: Up to 22 October 2020
Strategy: #1 AND #2 AND #3 AND #4 AND #5 AND #6
#1… cancer OR neoplasm OR tumor OR malignancy OR carcinoma
#2… Death OR dying OR die
#3… Place OR location OR site
#4… palliative OR hospice OR terminal OR “End of life” OR supportive OR “Terminally ill”
#5… choice OR prefer OR decision OR wish
#6… Hospice OR Hospital OR Home

### Assessment of the Risk of Bias

After reviewing the purpose of the studies and inclusion criteria, a total of 27 studies in terms of quality was separately evaluated by two researchers (A.F. and S.B.). All disagreements were resolved by discussion and agreement between the two researchers. In case of disagreement between the researchers, a third person was used as a judge. The quality of these articles was assessed using the Newcastle-Ottawa Edited Scale (Observation Studies Version) (Wells et al., 2000). In this scale, articles were assessed based on 4 criteria including representativeness of the samples, sample size (non-respondents, and measurement tool), comparability (a section including review of confounders and other influencing factors), and results (from two aspects: Assessment of the outcome and statistical test) were studied. Based on the Newcastle-Ottawa scale, articles were rated from zero (weakest study) to 10 (strongest study). For data maintenance, studies with a score lower than the mean score (less than a score of 4) were considered low quality. None of the 27 studies were excluded due to low quality.

### Data Extraction

Data extraction was separately conducted by two researchers A.F. and S.B. using a researcher-made form. Initially, an article was assessed as a pilot with this form; then was done for other articles. Each researcher used a data extraction form for their articles and the two lists were compared. All disagreements between the researchers were resolved by mutual agreement. In case of disagreement between the researchers, a third person was used as a judge and the result was reported after general agreement. Data related to the author, year, place of study, year of study, sample size, study design, preferred place of death (Home, Hospital, Hospice), and study quality was extracted. Using this form, the preferred place of death of patients with cancer was extracted and the results of the studies were classified into different factors.

### Statistical Analysis

Meta-analysis was performed using “meta” command in STATA 16 software. According to the existence of heterogeneity between studies, data were pooled using a random effects model by dersimonian- larid method. The heterogeneity of studies was assessed by the Cochran Q statistic. We planned to test the statistical heterogeneity with the Q test (χ2, I2, and Tau-squared statistics). The index I2 was interpreted by the following guide (Deeks et al., 2019).

(I2: 0–40; mild, I2: 40–70; moderate, I2: 70–90; sever, and I2; 90–100; highly sever).

The findings were considered heterogeneous if the *P* value was <0.1. Moreover, I2 was utilized to provide a model of the degree of inconsistency between the results of the studies. A value of 0% indicated no observed heterogeneity, whereas larger values showed increasing heterogeneity. Moreover, Egger test was used to determine publication bias in results. In case of encountering a duplicate article, only one duplicate study was used in the relevant composition. If the data is a graph, Web plot Digitizer software at: https://apps.automeris.io/wpd, was used, and if it is not a graph, corresponded with the responsible author. If no response was received within three different time intervals of 10 days, the data related to the initial objectives of the study were deleted.

## Results

### Studies Identified

**Figure 1:**
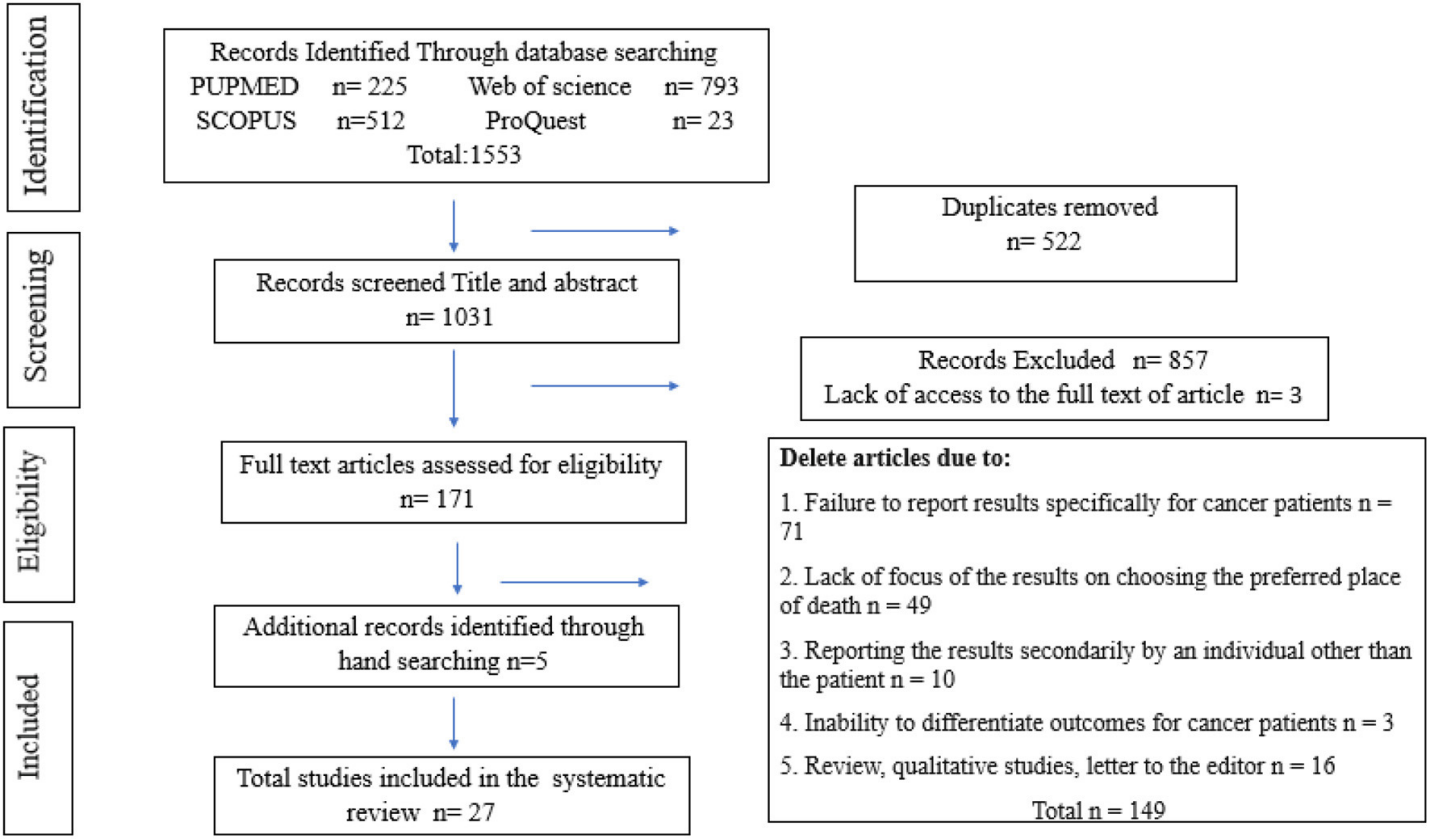
PRISMA flowchart.

One thousand five hundred fifty-three articles were found after searching. Endnote software version 8, (End Note. Thomson Reuters, X8) was used to organize information. Using the mentioned software and reviewing the title and abstract of articles, 522 duplicate articles were removed. Then, the title and abstract of 1031 articles were reviewed by researchers (A.F) and (S.B). A total of 857 unrelated articles were deleted in accordance with the objectives of the study. At this stage, if a study is suspicious, the full text of the article was reviewed by the researchers. In the next step, a search was performed to access the full text of the articles, access to the full text of 3 articles was not possible, and finally, the full text of 171 articles was reviewed. By considering the inclusion and exclusion criteria in accordance with the objectives of the study, Articles due to lack of research results specifically for cancer patients (*n* = 71), Lack of focus on choosing the preferred place of death (*n* = 49), reporting secondary results by a person other than the patient (*n* = 9), review, qualitative studies, letter to the editor (*n* = 16), the inability to differentiate outcomes for cancer patients (*n* = 3) was eliminated. To ensure the retrieval of all articles, the list of sources of the final articles was also manually searched and 5 more articles were added to the final articles. Finally, 27 studies were finalized. The process of entering studies based on inclusion and exclusion criteria was shown in Figure 1.

### Articles' Descriptive Characteristics

Table 2 summarizes the characteristics of the studies selected for meta-analysis. In this table, the author, year of publication, sample size, study design, study time, study population, country, continent, and preferred place of death (home, hospital, hospice) were separately expressed for each study. The number of participants in all studies was 14,920. Based on the year, studies were conducted in different years, the number of which is based on the year of study, including 1995 to 2005: 13 studies, 2006-2010: 5 studies, and 2011-2020: 7 studies. In 2 studies, the time of the study was not mentioned. By continent, 11 studies were conducted in Asia, 4 in the Americas, 8 in Europe, 3 in Africa, and 1 in Australia. Both men and women participated in all studies. According to the study design, 15 studies were cross-sectional, 11 were cohort studies and 1 was case-control studies. Of the 27 studies that reported Home: 55% of cancer patients with a safe interval [95% CI (61-49)] listed home as the preferred place of death; of the 21 studies that reported Hospital, 17% of patients with a confidence interval [95% CI (12-23)] preferred hospital as their favored place of death, and of the 12 studies that reported Hospice, 10% of patients with a confidence interval [95% CI (8-13) preferred hospice centers as their preferred place of death.

**Table 2:** Characteristics and results of included studies.

^*^N	**Author Year**	**Continent country**	**Study design Study time Sample size**	**PPOD of Home** **N (%)**	**PPOD of Hospital** **N (%)**	**PPOD of Hospice** **N (%)**	^**^ NOS Score
1	Kern et al. (2020)	Europe Switzerland	Cross Sectional 2015–2016 *n*: 116	34	9	^**^NR	5
2	Portorani et al. (2020)	Asia Iran	Cross Sectional (Short report) 2018 *n*: 274	176	NR	NR	7
3	Sheridan et al. (2020)	Europe UK	Prospective Cohort 2004–2012 *n*: 453	184	80	82	8
4	Alsirafy et al. (2019)	Africa Egypt	Cross Sectional 2014–2018 *n*: 272	253	19	NR	8
5	Blanchard et al. (2019)	Africa Southern Africa	Prospective cohort 2016-2018 *n*: 191	127	NR	NR	7
6	Shen et al. (2018)	Africa South Africa	Cohort 2016–2017 *n*: 221	127	51	4	7
7	Skorstengaard et al. (2017)	Europe Denmark	Cross Sectional 2013–2015 *n*: 81	29	1	33	7
8	Howell et al. (2017)	Europe UK	Cohort 2005–2010 *n*: 142	65	40	24	7
9	Gu et al. (2015)	Asia China	Prospective cohort 2007–2012 *n*: 522	280	204	NR	7
10	Guerriere et al. (2015)	America Canada	Prospective cohort 2010–2012 *n*: 302	185	^***^NR	NR	8
11	Chen et al. (2014)	Asia Taiwan	Cross Sectional 2003–2004 *n*: 2034	1114	483	53	6
12	Lee et al. (2014)	Asia Taiwan	Cross Sectional 2009–2011 *n*: 439	212	85	NR	7
13	Aoun and Skett (2013)	Australia Australia	Cross Sectional 2009–2010 *n*: 43	19	4	11	7
14	Jeurkar et al. (2012)	America USA	Retrospective Cohort 2000–2008 *n*: 5837	4336	35	254	8
15	Ikezaki and Ikegami (2011)	Asia Japan	Retrospective Case control 2005 *n*: 1664	810	207	NR	8
16	Ishikawa et al. (2013)	Asia Japan	Cross Sectional 2011 *n*: 258	123	NR	NR	7
17	Blaney et al. (2011)	Europe Ireland	Retrospective Cohort 2007 *n*: 283	173	36	41	7
18	Alonso-Babarro et al. (2011)	Europe Spain	Prospective cohort 2004–2006 *n*: 380	182	NR	NR	7
19	Nakamura et al. (2010)	Asia Japan	Cross Sectional 2005–2006 *n*: 92	37	18	NR	7
20	Stajduhar et al. (2008)	America Canada	Cross Sectional 2001–2003 *n*: 56	28	17	NR	7
21	Hsieh et al. (2007)	Asia Taiwan	Cross Sectional NR *n*: 46	34	12	NR	8
22	Kui et al. (2005)	Asia Korea	Cross Sectional NR *n*: 371	175	118	54	7
23	Tang et al. (2005)	Asia Taiwan	Cross Sectional 2003–2004 *n*: 559	341	135	11	7
24	Thomas et al. (2004)	Europe UK	Cohort 2000–2002 *n*: 41	10	0	8	8
25	Tang and McCorkle (2003)	America USA	Prospective cohort 2001–2002 *n*: 127	111	3	10	7
26	Gyllenhammar et al. (2003)	Europe Swedish	Cross sectional 1999 *n*: 221	81	NR	NR	7
27	Lee and Pang (1998)	Asia Singapore	Cross sectional 1995 *n*: 44	23	15	NR	7

* *Number*.

** *Newcastle—Ottawa Quality Assessment Scale*.

*** *No Report*.

### Prevalence Preferred Place of Death

#### Prevalence of Home-Based PPOD

**Figure 2:**
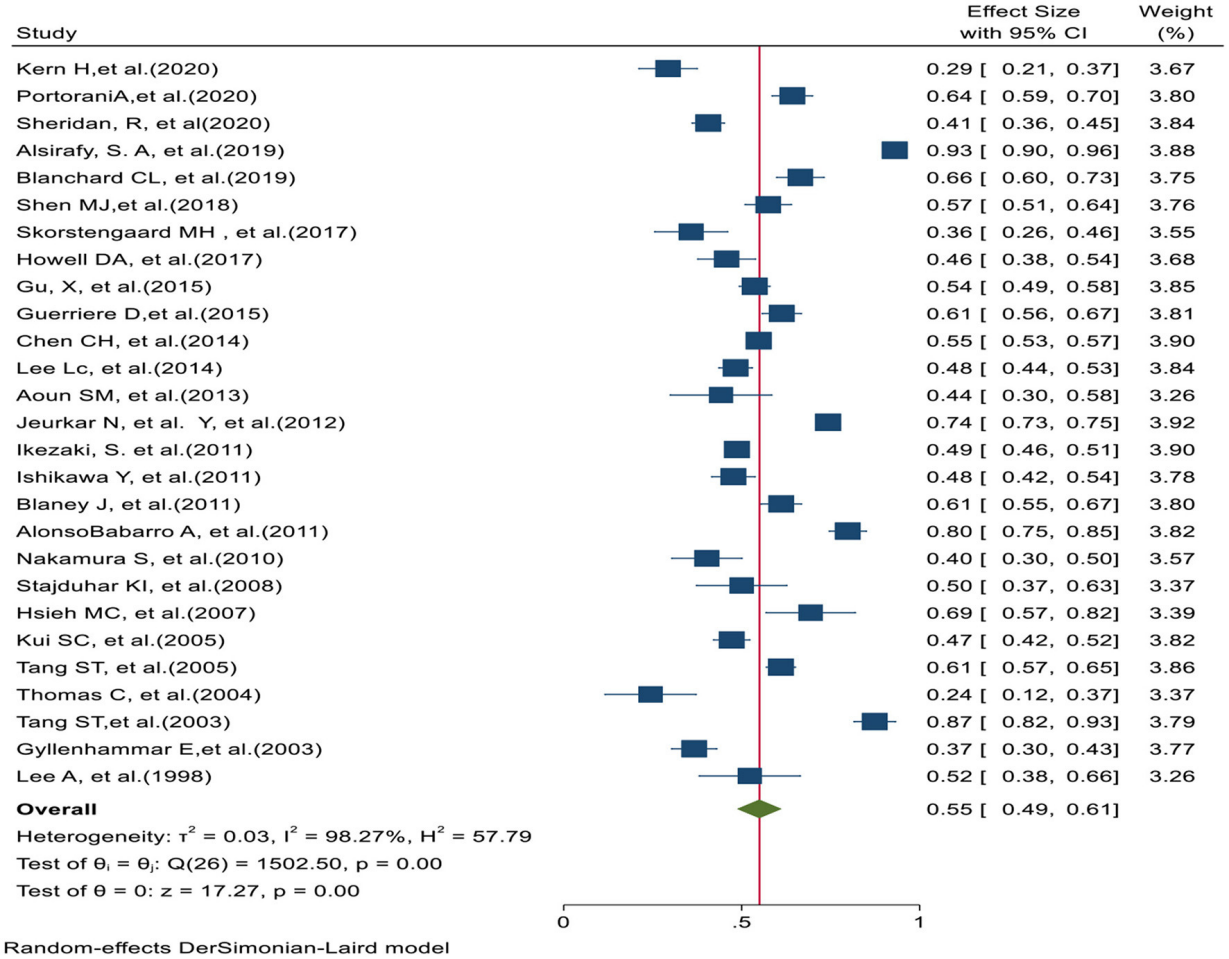
Forest plot for the prevalence of Home-based PPOD.

In 27 studies, the preferred place of death in home was reported. Based on the prevalence Home PPOD test of heterogeneity (χ2 = 1502.50, chi-square DF = 26, *P* ≤ 0.001) and heterogeneity indices [I2 = 98.27% and tausquared = 0.025], we used a random-effects model to calculate the prevalence. The pooled prevalence of Home PPOD in these studies was 55% [95% CI (49–61)]. Moreover, Egger test (z = −2.17, *P* value = 0.029) showed that there was publication bias in results. Figure 2 shows the preferred place of death at home in all studies.

#### Prevalence of Hospital-Based PPOD

**Figure 3:**
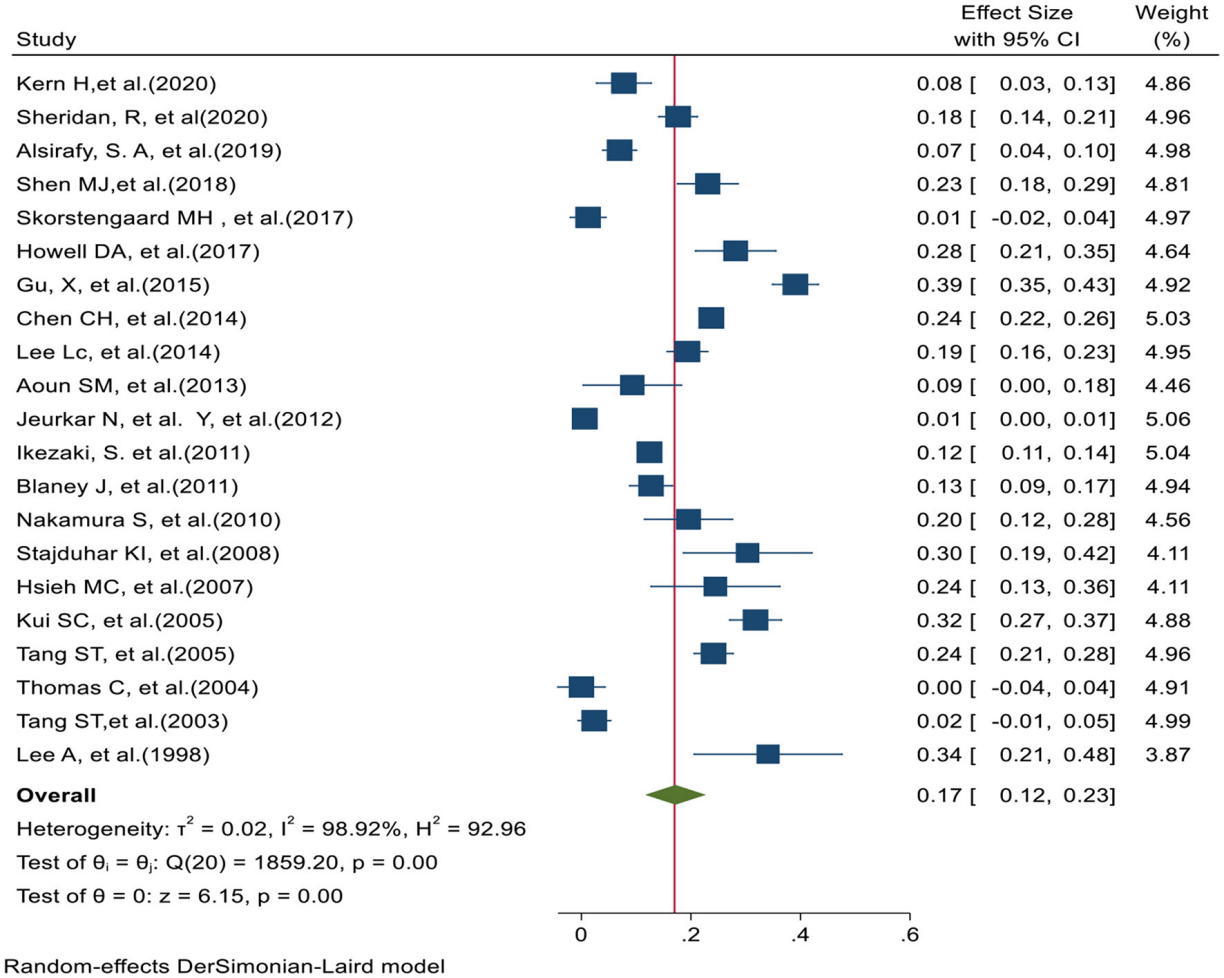
Forest plot for the prevalence of Hospital-Based PPOD.

In 21 studies, the preferred place of death in hospital was reported. Based on the prevalence Hospital PPOD test of heterogeneity (χ2 = 1859.20, chi-square DF = 20, *P* ≤ 0.001) and heterogeneity indices [I2 = 98.92% and tausquared = 0.015], we used a random-effects model to calculate the prevalence. The pooled prevalence of Hospital PPOD in these studies was 17% [95% CI (12–23)]. Moreover, Egger test (*z* = 2.32, *P* value = 0.020) showed that there was publication bias in results. Figure 3 shows the preferred place of death at hospital in 21 studies.

#### Prevalence of Hospice-Based Preferred Place of Death

**Figure 4:**
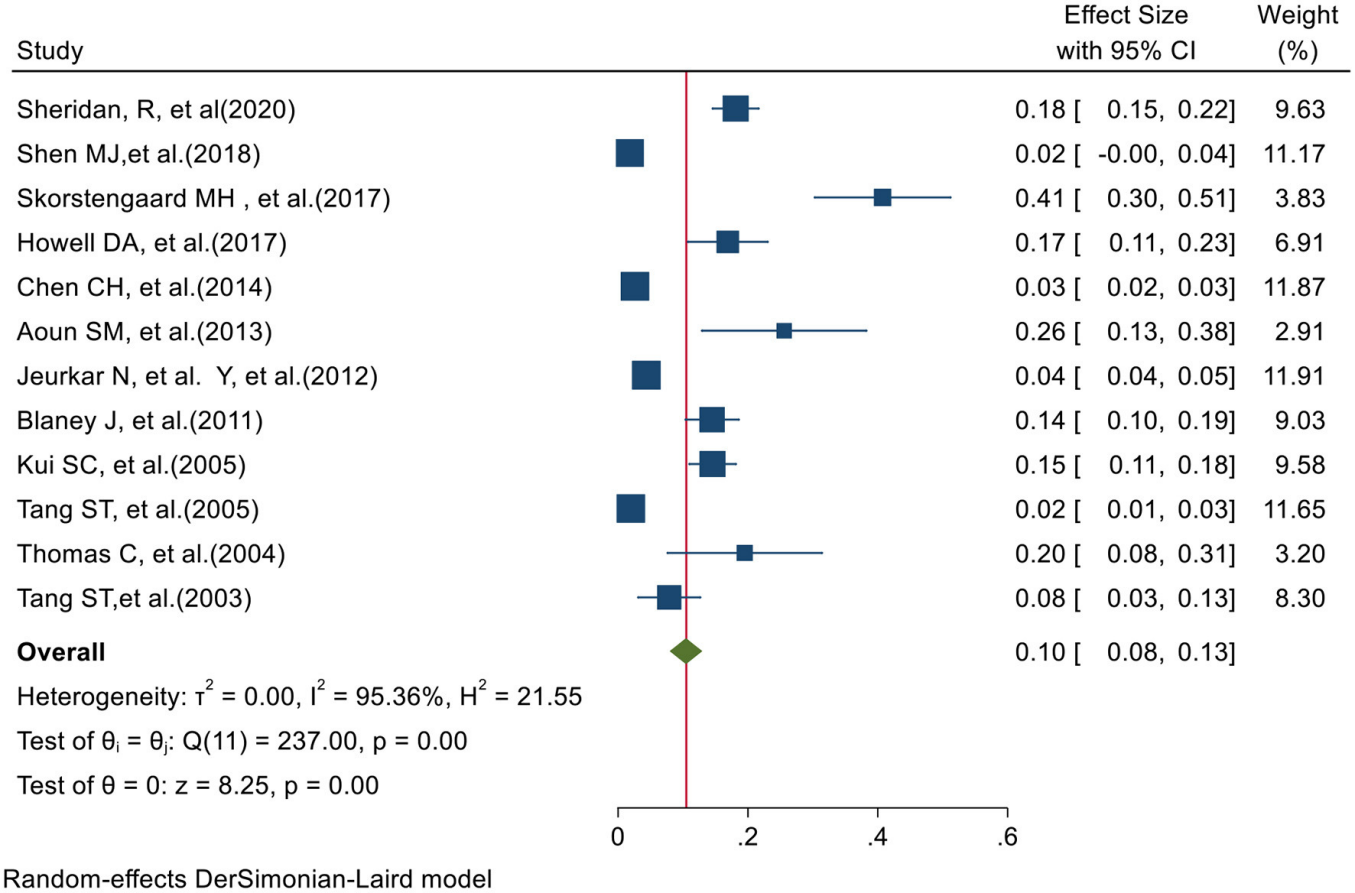
Forest plot for the prevalence of Hospice-based PPOD.

Figure 4 shows the preferred place of death at hospice in 12 studies. Based on the prevalence PPOD Hospice test of heterogeneity (χ2 = 237.00, chi-square DF = 11, *P* ≤ 0.001) and heterogeneity indices [I2 = 95.36% and tausquared = 0.001], we used a random-effects model to calculate the prevalence. The pooled prevalence of PPOD Hospice in these studies was 10% [95% CI (8–13)]. Moreover, Egger test (*z* = 7.39, *P* value = *P* ≤ 0.001) showed that there was publication bias in results. Figure 4 shows the preferred place of death at Hospice in 21 studies.

### Factors Influencing the Choice of Preferred Place of Death in Cancer Patients

Various studies have identified different contributing factors to the preferred place of death for cancer patients (Ikezaki and Ikegami, 2011). These factors play an important role in choosing the preferred place of death in cancer patients. Studies have been considered influential the factors such as early diagnosis, marital status, early referral to palliative care specialists, socioeconomic status, age (Bell et al., 2010), gender, place of residence (urban or rural), education, job status, and knowledge of the concept of death in choosing the preferred place of death (Foreman et al., 2006). According to the factors in different studies, factors affecting the choice of preferred place of death in cancer patients were divided into Three groups (Demographic characteristics, disease-related factors and psychosocial factors).

### Demographic Characteristics

In 3 studies “age” (Blaney et al., 2011; Jeurkar et al., 2012; Blanchard et al., 2019), in 2 studies “gender” (Kui et al., 2005; Sheridan et al., 2020), in 1 study “area of residence” (Gu et al., 2015), in 2 studies “level of education” (Chen et al., 2014; Gu et al., 2015), in 1 study “race” (Jeurkar et al., 2012), in 3 studies “the status of life,” (living alone or with other family members) (Gyllenhammar et al., 2003; Guerriere et al., 2015; Gu et al., 2015) in one study, “the initial place of patient care” (Jeurkar et al., 2012) was significantly associated with the choice of the preferred place of death in cancer patients.

In the study by Blaney et al. (2011) younger patients were more likely to choose the hospital. In the study by Jeurkar et al. (2012) younger patients chose home more as the preferred place of death, in the study by Blanchard et al. (2019) older patients chose home more as the preferred place of death. In a study by Kui et al. (2005) women were less likely than men to choose the home as a preferred place of death, and In a study by Sheridan et al. (2020) women were more likely to discuss on preferred place of death. In a study by Gu et al. (2015) and Chen et al. (2014) patients with a lower level of education chose home as their preferred place of death.

In a study by Gu et al. (2015) patients living in rural areas chose home as their preferred place of death. Also, in this study, patients living with family members were more likely to choose the home, unlike the study of in the study by Guerriere et al. (2015) patients living alone were less likely to choose home as their preferred place of death. In the study by Jeurkar et al. and Gu et al. married people were more likely to choose home as their preferred place of death (Jeurkar et al., 2012). In a study by Jeurkar et al. (2012) whites' patients were more likely than blacks to choose the home as their preferred place of death.

### Disease-Related Factors

In 3 studies “type of cancer” (Chen et al., 2014; Howell et al., 2017; Blanchard et al., 2019), 2 studies “time of diagnosis” (Chen et al., 2014; Gu et al., 2015) 1 study “prognosis of the disease” (Jeurkar et al., 2012), 1 study of patient symptoms such as pain (Blanchard et al., 2019), 1 study “medical treatment”, 2 studies “disease awareness” (Gyllenhammar et al., 2003; Blanchard et al., 2019), 1 study of “satisfaction with treatment” (Gu et al., 2015), 1 study “the functional status” (Jeurkar et al., 2012) of patients was a significant related to the choice of preferred place of death in patients with cancer.

In the study of Chen et al. (2014) patients with liver and pancreatic cancer, and in the study of Blanchard et al. (2019) patients with lung or breast cancer were more likely to choose home as their preferred place of death.

In the study by Blanchard et al. (2019) patients who knew their prognosis were less likely to choose home as their preferred place of death. In the study by Jeurkar et al. (2012) patients with severe to moderate pain were less likely to choose home as their preferred place of death; Also in this study, patients with better and more independent functional status were less likely to choose the home, and patients who used morphine to relieve pain were more likely to choose home as their preferred place of death (Blanchard et al., 2019). In the study by Gyllenhammar et al. (2003) and Blanchard et al. (2019) people who were aware of their illness were more likely to choose home as their preferred place of death. In the study by Gu et al. (2015) patients who were satisfied with their treatment status and patients who were in the poor physical condition and survived for more than 6 months from the diagnosis were more likely to choose home as their preferred place of death.

### Psychosocial Factors

Psychological factors influencing the preferred place of death in cancer patients was including not imposing burden on family and other caregivers (Lee and Pang, 1998; Kui et al., 2005; Tang et al., 2005; Yamagishi et al., 2012), Patients' level of anxiety (Skorstengaard et al., 2017), awareness of the incurability of the disease (Gomes et al., 2015), participation or non-participation of social workers (Kern et al., 2020), Being with the family in the last days of life (Kui et al., 2005), having independence in doing daily tasks at the end of life (Tang et al., 2005, 2006), environmental security (Lee and Pang, 1998) and intimacy and emotional connection with family members (Lee and Pang, 1998).

## Discussion

The importance of patients' preferences is considered an important result of palliative care, so it is important to understand the preferred place of death and the factors that affect it in end stage cancer patients. Because the choice of this place is directly related to the spiritual and physical peace of patients and their families (Cabañero-Martínez et al., 2019). Therefore, this systematic review and meta-analysis study was performed to investigate the preferred place of death and the factors affecting it in adult patients with cancer.

Recent studies have also shown that preference for place of care and place of death is not a fixed concept and can change over time through discussion between health care professionals and patients (Butow et al., 1997; Munday et al., 2009). Of the 27 studies that reported Home: 55% of cancer patients with a safe interval [95% CI (61–49)] listed home as the preferred place of death; of the 21 studies that reported Hospital, 17% of patients with a confidence interval [95% CI (12–23)] preferred hospital as their favored place of death, and of the 12 studies that reported Hospice, 10% of patients with a confidence interval [95% CI (8–13) preferred hospice centers as their favored place of death. Results of the study by Jeurkar et al. (2012) show that in the United States, of 5,837 patients under study, about 56.5% with a confidence interval [95% CI (1.77–2.76)] of patients preferred to die at home (Jeurkar et al., 2012). In the study by Ikezaki and Ikegami (2011) on cancer patients in Japan, half of the patients and 42% of families preferred to die at home, when the patient and family preferences were different, it was the patient who preferred to die at home (Ikezaki and Ikegami, 2011). Another study In Taiwan, conducted by Chen et al. (2014), shows that more than half of the participants (54.7%), preferred to die at home (Chen et al., 2014). The results of a meta-analysis by Suzanne Rainsford et al. show that most patients reported a preferred place for care and death in the hospital and at home (Rainsford et al., 2016). Another systematic study reveals that home is the preferred place of death for most cancer patients worldwide (Bell et al., 2010). Debra A Howell et al. in a systematic study and meta-analysis show that Debra A Howell et al. (2010). In a systematic study and meta-analysis show that most patients with hematologic malignancy die in hospital more than twice as often as patients with other cancers. Given that the house is usually considered as their preferred place of death. If the patient prefers to die at home, he or she should be respected as much as possible, for example, some patients with moderate to severe pain can stay home with effective pain management. The patient may not be in a position to express his or her wishes, and the family's preference and capacity for care should be considered (Leff et al., 2000; Jack, and O'BRIEN, 2010; Silveira et al., 2010). In addition, the home may not have enough facilities to meet the needs of the dying person, people who can provide end-of-life care, and the resources of the health care system (Gomes and Higginson, 2008). The results of a study by Vidal et al. (2020) show that a significant number of patients have no preference or prefer to die in hospital, especially in patients who have already examined home care, have financial and social problems, or have uncontrolled physical and mental symptoms and distress. In many countries, such as the United Kingdom, Japan, South Korea, Greece, and Italy, the number of deaths at home is declining, and in some other countries, such as the United States and Canada, guidelines have been developed to reduce deaths at home (Higginson et al., 2013). Therefore, health care professionals should provide the patient and family with prognostic information and discussions about end-of-life care to facilitate their understanding of the patient's preferred place of death.

In the second part of the study, the factors related to the preferred place of death were investigated. Due to the lack of similarity between different studies, it was not possible to perform meta-analysis in this dimension, and the results have been reported qualitatively and do not necessarily indicate a specific direction and cause. Because each person's preferences are unique and influenced by a variety of factors, identifying priority predictors of the preferred place of death is useful for understanding how patients make decisions about where to live at the end of their lives. But the results of studies have mentioned many different factors. The present study reported the effective factors in the preferred place of death of cancer patients in two groups of demographic characteristics, disease-related factors. Disease-related factors highlight the issue of palliative care time. Patients in the more advanced stages of the disease with uncontrolled mental, physical, and social symptoms may die less at home because they have multiple treatment options, even in the advanced stages of the disease, and the responsibility of caring for family members is less. In most individual factors, the preferences, desires, and inclinations of the patient play an important role in achieving the place of death. In a systematic study, other factors influencing the preferred place of death in the two groups of maintaining patient individuality include demographic variables (relatively stable and unchangeable characteristics in patient identity), personal factors reflecting patients' beliefs, desires, and internal resources for adaptation, and underlying environmental factors (Gomes and Higginson, 2006). The results of diffusion bias according to Egger test in three death places were significant in terms of patient (home, hospital, and hospice) preferences.

Regarding the psychological factors affecting the preferred place of death, researchers report severe heterogeneity and the results are qualitatively reported, so the evidence was inconclusive. According to a study (Lee and Pang, 1998; Tang et al., 2005, 2006), intimacy, emotional connection with the family and having independence in doing daily tasks at the end of life are among the psychological factors affecting the preferred place of death. Solomon et al. reported in 2013 that examining family relationships emotionally, from the patient and family member's perspective, may enrich their understanding and ability to help patients die at home (Solomon and Hansen, 2015). Support from family members may require a good family relationship and mutual trust. Qualitative findings suggested that caregivers commit to providing care and to address the patient's preference to be at home, to then become aware of the complexities involved. Victoria Turner et al. showed the main factor affecting access to preferred place of death was social support; people with fewer informal carers were less likely to die in their preferred location. This highlights the importance of good communication of preferences and concerns between patients and caregivers throughout the process, and the need for practical and emotional support to caregivers, to meet the patient's preference when possible and to minimize the risk of difficult bereavement for caregivers (Gomes et al., 2013). Moreover, human factors such as social support and career resilience, plus the availability of resources such as care staff and hospice beds, as important factors in achieving preferred place of death (Turner and Flemming, 2019). Other sociological factor is Patients' level of anxiety. The participants in Pradilla study that experienced emotional symptoms such as depression and anxiety preferred dying in a health care environment. Their assumption is that these people are more prone to seek and need professional assistance (Pradilla et al., 2011). This stresses the role of the mental health professional when taking care of a terminal patient, as reported in the review by Gibson et al. (2006). Also, helping the next of kin to be aware of the impending death may increase the chance to die at home and based on preferred place (Lee et al., 2014). For a better hospice care service, it is essential to inquire patients or their relatives on preferred place of death while concerning the influences of other factors. Therefore, identifying the factors affecting the preferences of patients in the end stages of life and their families is a necessity that should be considered.

In our study, heterogeneity levels were calculated using Q and I2 tests, the level of which was reported to be high in terms of home death place (I2 = 98.27%), hospital (I2 = 98.92%), and hospice (I2 = 95.36%), respectively. One of the reasons for the increase in the level of heterogeneity can be the combination of different studies with different sample sizes. In the present study, the lowest sample size consisted of 41 samples and the highest sample size consisted of 5,837 samples.

## Advantage and Limitations

In our study, priori registration in PROSPERO system based on the principle of comprehensiveness and quality, data combination and investigation of the amount and possible causes of heterogeneity, as well as more inclusive search based on the use of synonymous detection systems Thesaurus Mesh and, Emtree, examining large databases such as PubMed, Scopus, web of science, ProQuest with extensive search time, using the opinion of experts, without time and space limitations, this systematic review can be compared to previous studies have a more comprehensive review of initial studies in the field. In the second part of the study, the factors related to the preferred place of death were investigated. Due to the severe heterogeneity between studies in this dimension, the results were qualitatively reported, which is one of the limitations of this study. Given that more than half of patients chose home as their preferred place of death, it is suggested that future studies on cost-effectiveness and health resource allocation be devoted to home-based end-of-life care.

## Conclusion

Based on the results of the present study, more than half of cancer patients in the later stages of life prefer to die at home. Also, the factors affecting the individual's preferences in deciding to choose the preferred place of death have been classified into two groups of demographic characteristics, disease-related factors. Considering that one of the goals of palliative care and end-of-life care program is to increase the quality of life of patients and their families, for this reason, guided policies to ensure the death of patients in the desired place according to the patient's condition should be a priority. Therefore, health care professionals should be aware of the palliative care needs of patients.

## Data Availability Statement

The original contributions presented in the study are included in the article, further inquiries can be directed to the corresponding author.

## Author Contributions

AF and SB conceptualized and designed the study, retrieved data, and rechecked the data. MS, AV-A, AF, and SB conducted study analyses. AF, SB, and MR did the initial drafting. AF, MR, HA, MS, AV-A, and SB critically assessed the data and provided intellectual inputs. All members approved the final draft.

## Conflict of Interest

The authors declare that the research was conducted in the absence of any commercial or financial relationships that could be construed as a potential conflict of interest.

## Publisher's Note

All claims expressed in this article are solely those of the authors and do not necessarily represent those of their affiliated organizations, or those of the publisher, the editors and the reviewers. Any product that may be evaluated in this article, or claim that may be made by its manufacturer, is not guaranteed or endorsed by the publisher.
